# Human monoclonal anti-protective antigen antibody for the low-dose post-exposure prophylaxis and treatment of Anthrax

**DOI:** 10.1186/s12879-018-3542-6

**Published:** 2018-12-10

**Authors:** Qi Tang, Siping Xiong, Xudong Liang, Xingwang Kuai, Yiwen Wang, Changjun Wang, Zhenqing Feng, Jin Zhu

**Affiliations:** 1 Key Laboratory of Antibody Technique of National Health and Family Planning Commission, Nanjing Medical University, Nanjing, 210029 China; 2 Huadong Medical Institute of Biotechniques, No.293 Zhongshan Dong Road, Nanjing, 210002 China; 3 National Institute for Communicable Disease Control and Prevention, Chinese Centre for Disease Control and Prevention, Beijing, 102206 China

**Keywords:** Anthrax, Full human antibody, Protective antigen (PA), Lethal toxin, Neutralising

## Abstract

**Background:**

Disease caused by *Bacillus anthracis* is often accompanied by high mortality primarily due to toxin-mediated injury. In the early disease course, anthrax toxins are secreted; thus, antibiotic use is limited to the early stage. In this regard, antibodies against the toxin component, protective antigen (PA), play an important role in protecting against anthrax. Therefore, we developed PA21, a fully human anti-PA immunoglobulin G monoclonal antibody.

**Methods:**

Combining human Fab was screened from a phage library with human heavy constant regions. Enzyme-linked immune sorbent assay, Western blot analysis and immunoprecipitation test evaluated the binding ability of PA21. Moreover, the affinity and neutralizing activity of the antibody was detected in vitro while the protective effectiveness in 60 rats was also examined in vivo.

**Results:**

The Fischer 344 rats challenged with the lethal toxin can be protected by PA21 at a concentration of 0.067 mg/kg. All six rats remained alive although PA21 was injected 24 h before the toxin challenge. PA21 did not influence the binding of PA to cell receptors and that of a lethal factor to PA.

**Conclusion:**

The PA21 monoclonal antibody against PA can be used for emergency prophylaxis and anthrax treatment.

## Background

*Bacillus anthracis*, a Gram-positive bacterium, causes illness and death in animals and humans [1]. Given the historical weaponization of this agent since World War II, the bacterium is considered a biological threat, especially after the anthrax letter attacks in 2001 in the United States [2]. The anthrax toxin, which is a mixture of three secreted proteins, plays a crucial role in the death of humans and animals affected by anthrax [3]. The anthrax toxin is composed of a protective antigen (PA), a lethal factor (LF) and an edema factor (EF). These three proteins are individually nontoxic, but PA combines with LF and EF can form the lethal toxin (LT) and edema toxin (ET), respectively [4, 5]. PA mediates the cellular uptake of LF and EF. LF is a zinc-dependent metalloprotease that inactivates mitogen-activated protein kinase kinases [6, 7], whereas exhibits calmodulin-dependent adenylyl cyclase activity that increases intracellular cAMP levels [8, 9]. Before EF or LF translocation to the cytosol, PA must firstly bind to the anthrax toxin receptors on cell surface [10, 11]. After binding, PA can be cleaved by a cell-surface-associated furin to generate the active molecule PA63. Then PA63 can form a heptameric prepare complex which would bind EF/LF [12, 13]. Consequently, LF causes cell death and EF causes cell edema [14, 15].

In recent years, several recombinant monoclonal antibodies (mAbs) against PA have been shown to protect animals from anthrax toxin challenges [2, 16–18]. Many anti-PA mAbs have been developed from murine [19, 20] and human sources [1, 21, 22]. These antibodies contain a murine component that may be recognised by the human anti-mouse antibody. Moreover, several antibodies have been characterised from severely combined immunodeficient mice with transplanted human immune systems [23, 24]. However, these antibodies are difficult to obtain. Most of these antibodies also inhibit PA binding onto cell receptors, whereas others prevent LF binding to PA63, and some inhibit PA cleavage by furin. A small number of antibodies are known to affect PA heptamerisation. Given this information, we desire to construct a human antibody that thoroughly exerts neutralizing effectiveness in a distinct mechanism.

Herein, we report the development of an anti-PA immunoglobulin G (IgG) antibody derived from a human Fab phage library. This fully human antibody exhibited high affinity and promising protective ability, in vitro and in vivo.

## Methods

### Development of human anti-PA IgG

**Fig. 1:**
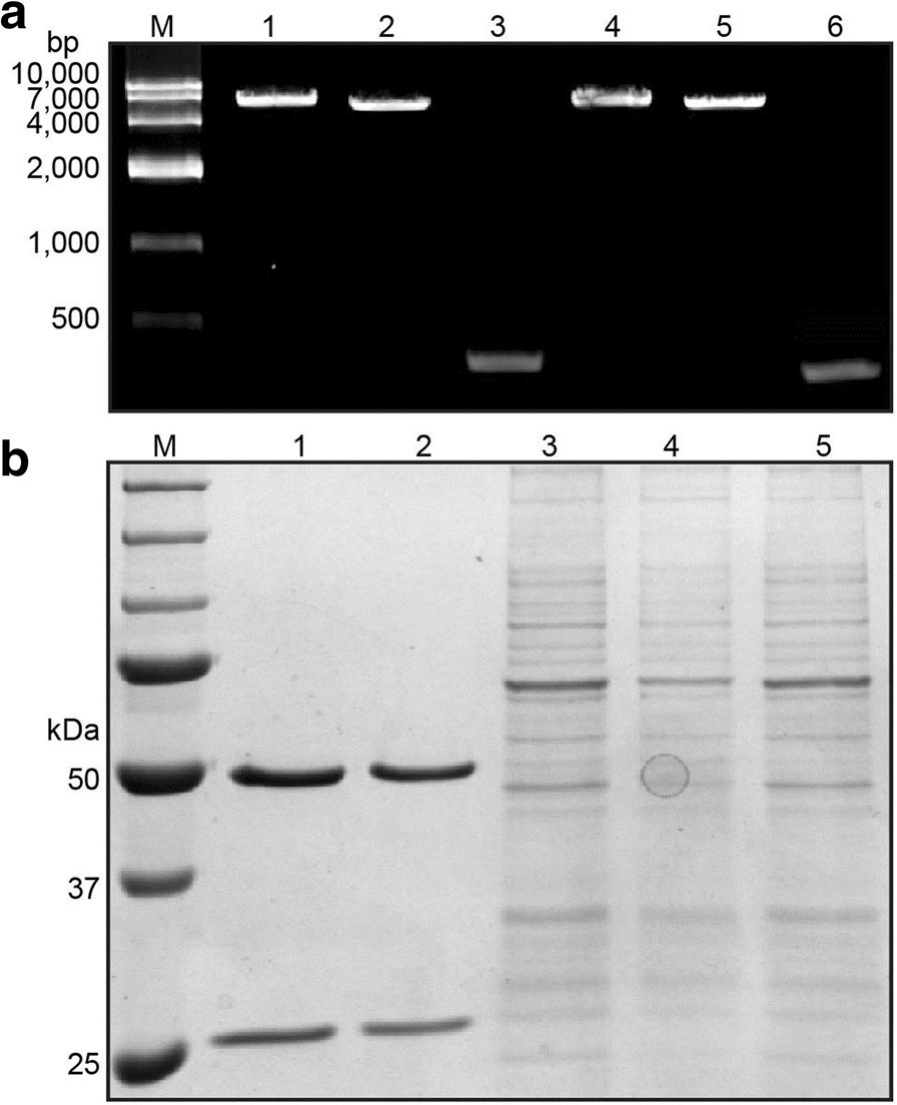
**a** Construction of PA21 expression vectors. M, marker; lane 1, expression vector for PA21 heavy chain; lane 2, linearised pTH; lane 3, variable region of PA21 Fab heavy chain; lane 4, expression vector for PA21 kappa chain; lane 5, linearised pTL; lane 6, variable region of PA21 Fab kappa chain. **b** Purification of PA21. M, marker; lane 1, PA21 antibody; lane 2, control human IgG; lane 3, PA21 (cell supernatant); lane 4, PA21 (flow through); lane 5, 293F cell supernatant

A human Fab-phage library, constructed as previously described, was employed to screen human Fab against PA [25]. This phage library, which was preserved by the key laboratory of antibody technique of National Health and Family Planning Commission, Nanjing Medical University, was titrated and 2 × 10^10^ phage clones were collected for first-round panning. The VCSM13 helper phage and the *E. coli* strain XL1-Blue and another *E. coli* strain, Top 10 F′, were used for Fab expression. After 7 rounds of panning, 45 single phage clones were randomly picked up and amplified to test for specific binding to PA83 by phage ELISA. Positive clones were defined when the ratio of sample OD450 versus the blank of was greater than 2.5. 19 clones with strongest binding to PA83 were analyzed by DNA sequencing and the best one was named as PA21.The clone PA21 Fab was selected for transformation to full human IgG. Recombinant IgG expression vectors were expressed in 293F cells. Then, the cell supernatant was purified with a HiTrapTM Pro. A HP column (GE, USA). The purified protein, control IgG, cell supernatant and flow through were separately analysed by 10% sodium dodecyl sulphate-polyacrylamide gel electrophoresis (SDS-PAGE) (Fig. 1).

### Enzyme-linked immunosorbent assay (ELISA)

ELISA was performed as previously described [22]. The plates were coated with PA83 antigen, and PA21 (each concentration for three duplicated wells) was added as the primary antibody. Absorbance values of PA21 at 450 nm were plotted using the GraphPad Prism software version 5.0 (GraphPad Software, Inc., La Jolla, CA, USA). The experiment was repeated for three independent times.

### Immunoprecipitation

Lysed PA83 bacteria were incubated with 5 μg PA21 at 4 °C, followed by the protocol described elsewhere [22]. A non-correlated human IgG was incubated with lysed PA83 as the negative control. The protein complexes were then isolated by10% SDS–PAGE gel. Then, the target band (~ 83 kDa) was subjected to the mass spectrum identification. The mass spectra were searched against the Swiss-Prot database using the MASCOT search engine (http://www.matrix science.com).

### Affinity and kinetic assay for antibody

The affinity and kinetics of the PA21 antibody was performed with the Biacore X100 System (GE, USA). PA83 antigen was immobilized on the surface of a CM5 sensor chip (GE, USA) at a concentration of 25 μg/mL in acetate buffer (10 mM NaAc, pH 4.5). The purified PA21 was diluted to different concentrations ranging from 5 to 80 nmol/L in running buffer (10 mM HEPES, 150 mM NaCl, 5 mM EDTA-Na2, 0.05% P20; pH 7.4). The experiment was then conducted according to the protocol of Biacore X100 System. Finally, the sensograms were evaluated using Biacore X100 evaluation software.

### In vitro LeTx neutralisation assay

The in vitro LeTx neutralisation assay was performed as described previously [26]. Briefly, J774A.1 cells were seeded in 96-well plates overnight. Ten-fold serial dilutions of LF was added in complete medium containing PA and PA21. The mixture was treated to the cells at the following final concentrations: LF, 0.01–10,000 ng/mL; PA, 0.1 μg/mL; and PA21, 4 μg/mL, triplicate wells for each concentration. The plates were then incubated for 3 h at 37 °C. Untreated cells and those treated with only LeTx were used as the controls. Cell viability was determined using the AQ assay (Promega, USA) in accordance with the manufacturer’s instructions. Three independent times were adminstered for the experiment.

### In vivo LeTx neutralisation assay

Female F344 rats weighing between 130 and 160 g were used for in vivo LeTx neutralisation assay. A total of 60 rats were randomly divided into 10 groups, with 6 rats in each group. Each rat in the 3 groups was injected via the tail vein with a mixture of PA + LF (LeTx) and different amounts (0, 5 or10μg) of PA21 antibody prepared in sterile PBS. Each rat was then administered with 300 μL of the mixture. The other rat groups were injected intravenously with different concentrations of the antibody at 5 or 10 mins after receiving an intravenous injection of LeTx (30 μg PA + 30 μg LF). Additionally, the remaining rats were injected with double the complete protective dose of the antibody (20 μg) to test the prophylactic ability of the antibody. The rats were inoculated with 20 μg antibody followed by LeTx administration after different periods ranging from 5 min to 30 h. After LeTx was injected, the animals were observed continuously for the first 8 h,16 h, throughout the second day and twice-daily for 1 week. At the end of the experiments, all rats were anaesthetized by CO_2_ and killed.

All the experiments that involved animals were performed in accordance with the protocols approved by the Institutional Animal Care and Use Committee of Nanjing Medical University, China.

### Competitive ELISA

Plates were coated with 100 μL PA63 antigen (2 μg/mL). After blocking, serial twofold dilutions of LF (each concentration for three duplicated wells) and 0.125 μg/mL PA21 were added to the wells, which were then incubated for 2 h at 37 °C. Then, ELISA was performed as described above. The experiment was repeated for three independent times.

### Western blot of J774A.1 cells

J774A.1 cells were previously bought from Shanghai Institute of Biochemistry and Cell Biology (Shanghai, China). J774A.1 cells were cultured in 24-well plates overnight. PA83(1 μg) and different amounts (1,10 or30μg per well) of PA21 were added to the wells. After 3 h incubation, the wells were washed thrice with PBST. Then, the cells were lysed with RIPA (Promab, China) for 30 min on ice. Cell lysates were separated by 10% SDS–PAGE. Then, Western blot analysis was performed as described above. The PA combined with cells was detected, and glyceraldehyde-3-phosphate dehydrogenase and combined PA21 were used as controls.

### Survival analysis

Kaplan–Meier analysis was used to evaluate the prognosis status. Survival data were then analyzed using the GraphPad Prism version 5 statistical analysis software. *t*-Test was performed to compare the mean survival time among groups. A two-tailed log rank test was used to determine the statistical significance of the differences among groups. A *P* value of < 0.05 was considered statistically significant.

## Results

### Specificity and binding affinity of PA21

**Fig. 2:**
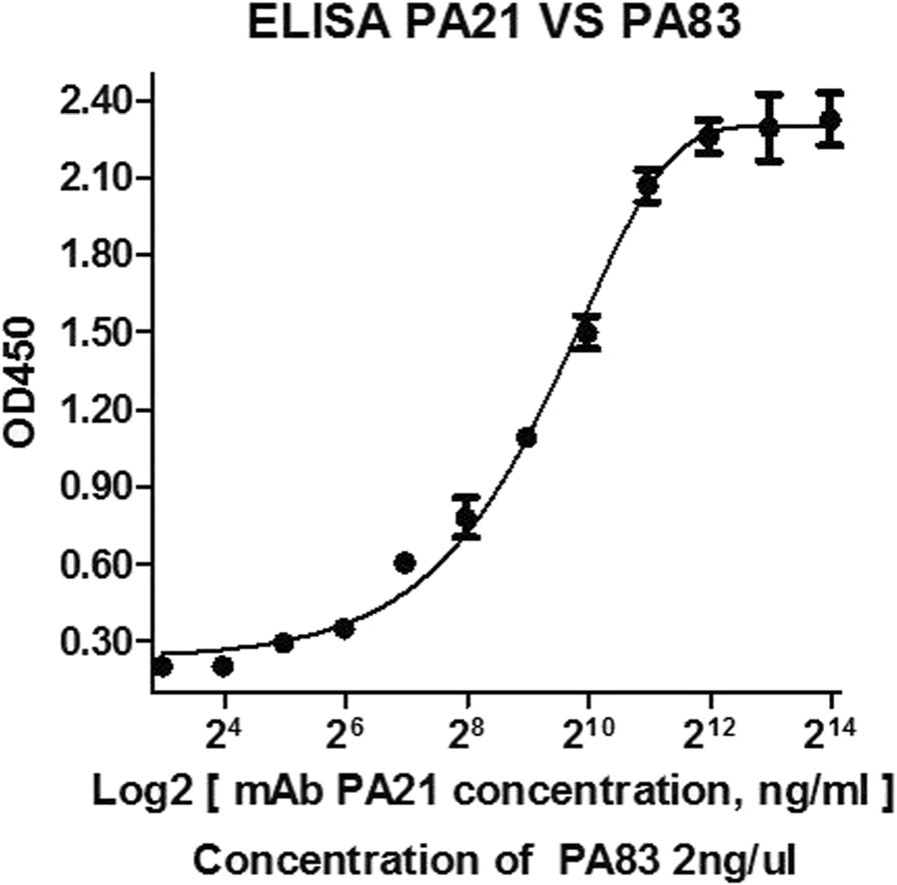
ELISA. PA83 was used to coat ELISA plates. The wells were then incubated with serial dilutions of PA21, and the bound antibody was detected by adding peroxidase-conjugated goat anti-human antibody followed by tetramethylbenzidine substrate. OD450 = optical density at 450 nm

**Fig. 3:**
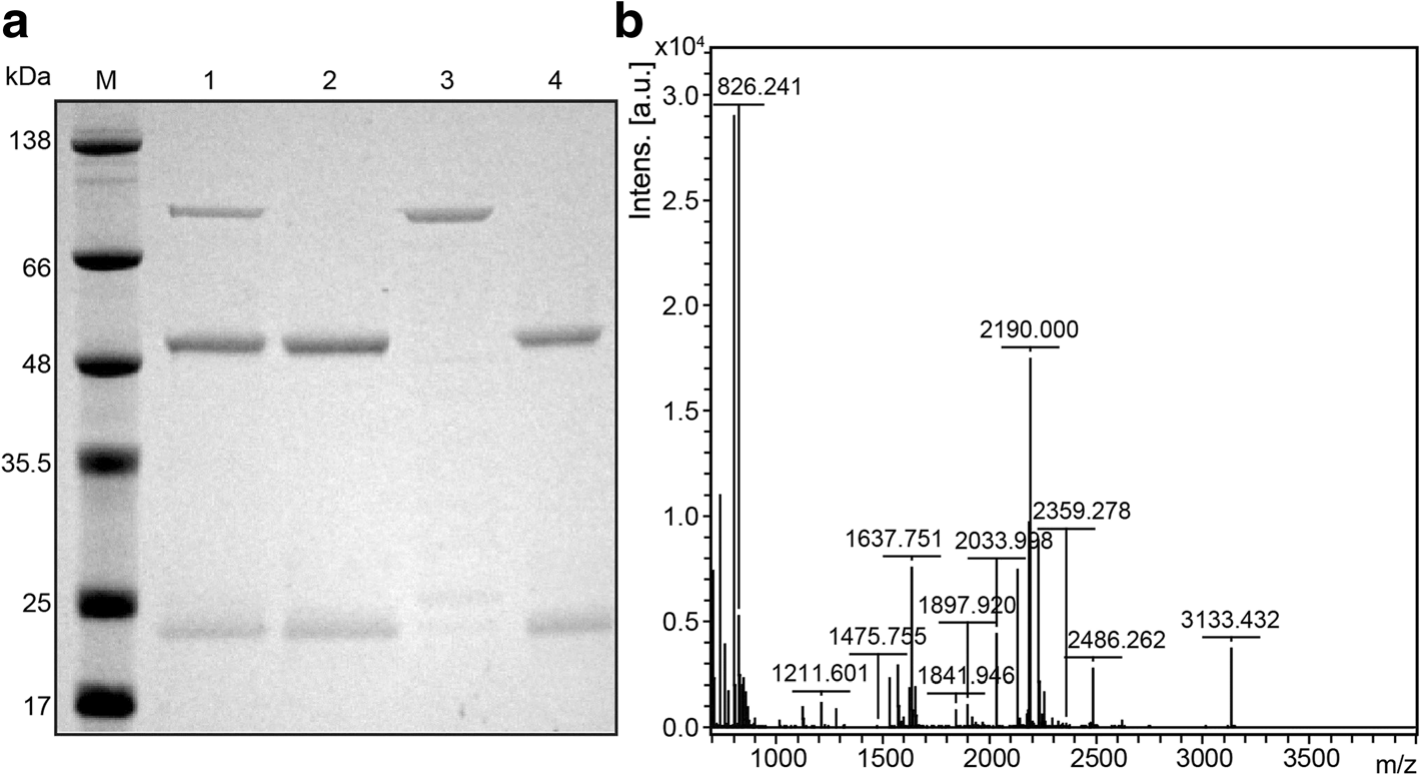
Immunoprecipitation (IP). **a** 10% SDS–PAGE of IP; M, marker; lane 1, PA21 + lysates of PA83 recombinant bacteria; lane 2, PA21; lane 3, PA83; lane 4, control antibody+lysates of PA83 recombinant bacteria. **b** MS spectra of fragment ions from the 83-kDa protein

**Fig. 4:**
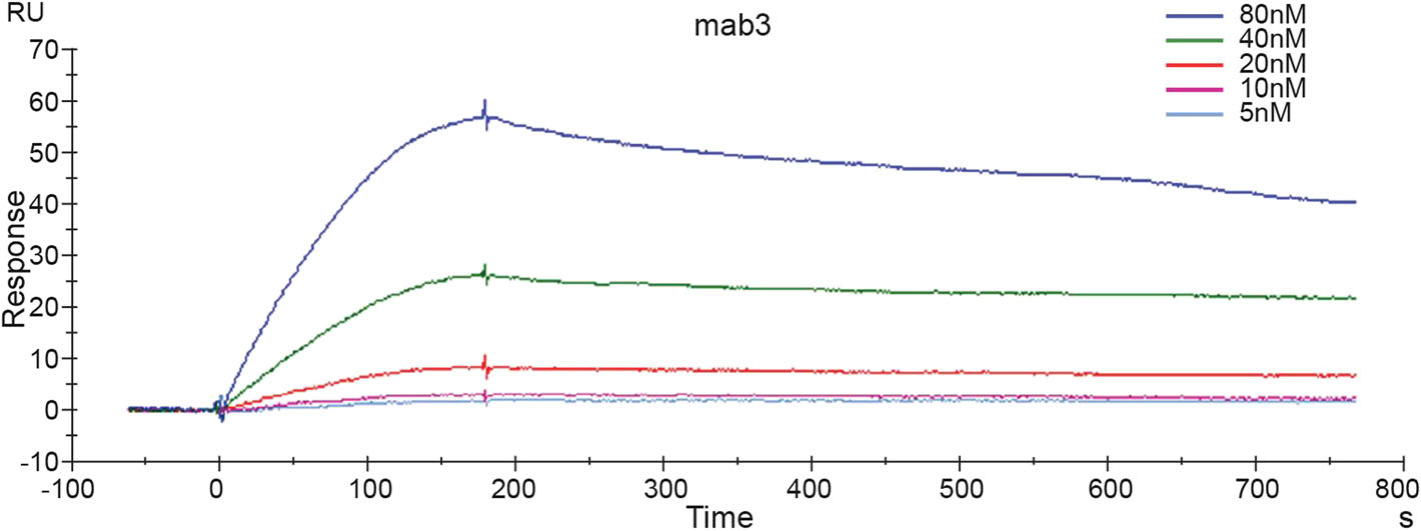
Affinity and kinetics assay. PA21 affinity and kinetics assays showed five curves with different concentrations of anti-PA IgG ranging from 5 nmol/L to 80 nmol/L; Kd = 1.0003 × 10–9 M with PA83 at 25 μg/mL

LISA was performed to test the binding sensitivity of PA21. PA21 identified PA83 in a concentration-dependent manner and detected PA83 even at the low concentration of 0.3125 μg/mL. Through the GraphPad Prism software, the concentration of PA21 and the absorbance at 450 nm were plotted to hyperbolic curves (Fig. 2). Then, through immunoprecipitation, a protein of approximately 83 kDa in size was detected by SDS–PAGE; the band was analyzed by mass spectra, compared with the Swiss-Prot database and analyzed for *B. anthracis* PA (Fig.3a and b). Furthermore, the equilibrium dissociation constant (K_d_) of PA21 was determined by BiaCoreX100 analysis for affinity test. Rate constants k_on_ and k_off_ were evaluated directly from the sensogram in the BiaCoreX100 and the values were7.486 × 10^5^/Ms. and 7.511 × 10^− 4^/s, respectively. Moreover, the K_d_ was deduced by the BiaCoreX100. One striking feature of PA21 is the slow off rate, which contributed to the high affinity of 1.003 × 10^− 9^ M (Fig. 4).

### In vitro LeTx neutralisation assay of PA21

**Fig. 5:**
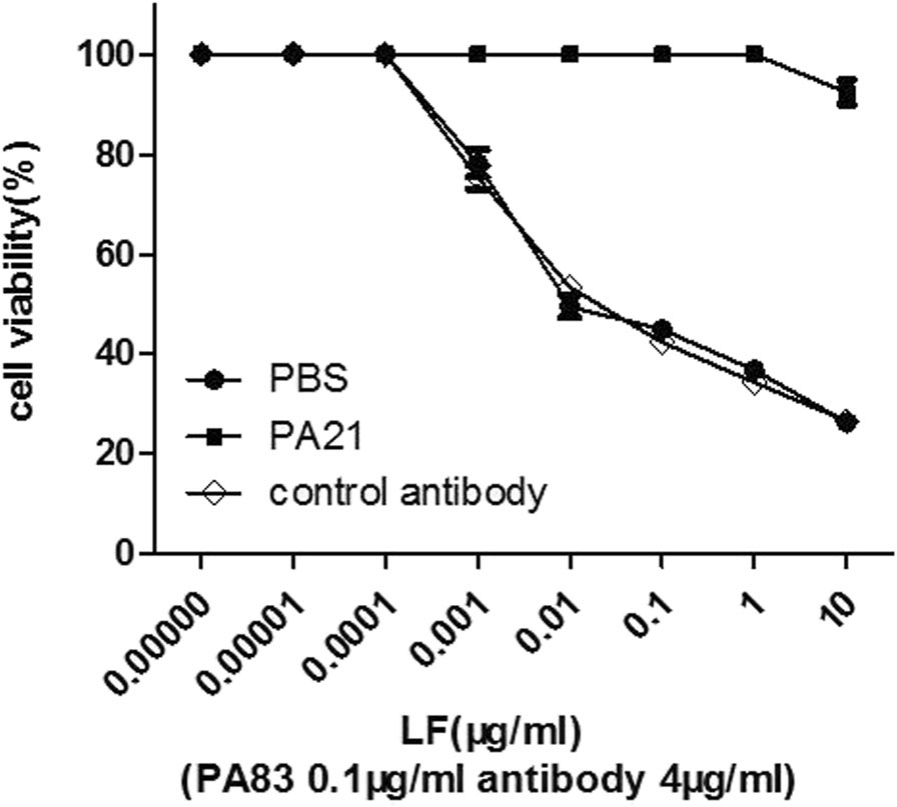
J774A.1 cell survival with PA21 treatment. Serially diluted LF was incubated with PA83 and different concentrations of antibodies for 3 h. Through the AQ assay, cell viability was determined and plotted as the survival percentage

J774A.1 cells were used to assess the protective ability of PA21 against LeTx. PA21, PA83and different concentrations of LF were added to cells simultaneously. The cell viability results showed that PA21 completely neutralised LeTx. PA21 protected more than 90% of cells when 10 μg/mL LF and 0.1 μg/mL PA83 were used, whereas the control antibody only protected 26% of cells (Fig. 5).

### Protective efficacy in vivo

**Fig. 6:**
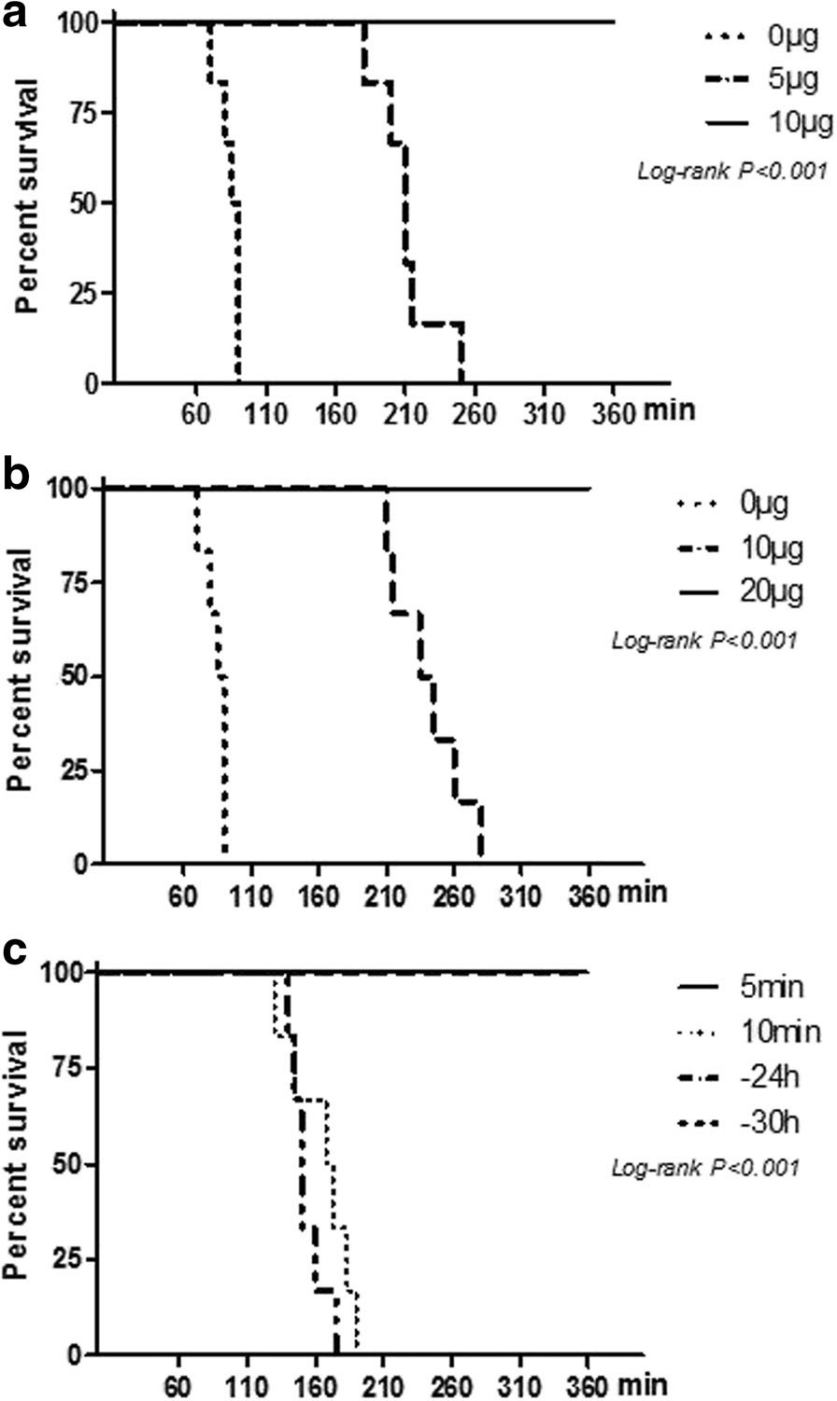
In vivo LeTx neutralisation assay in F344 rats. **a** LeTx and the antibody were simultaneously injected via the tail vein. 0 μg, 0 μg PA21 + 30 μg LeTx; 5 μg, 5 μg PA21 + 30 μg LeTx; 10 μg, 10 μg PA21 + 30 μg LeTx. **b** Different concentrations of the antibody were injected, and 30 μg LeTx was injected 5 min later via the tail vein. **c** For each rat, 20 μg antibody was injected before (− 24 h, − 30 h) or after (5 min, 10 min) LeTx

The in vivo test was performed in F344 rats. The antibody was injected via the tail vein simultaneously with, before or after LeTx injection. In the groups that underwent simultaneous injection, 10 μg PA21 protected all rats and 5 μg also prolonged the survival time relative to the control group (Fig. 6a). In the groups where the antibody was injected 5 min after LeTx, 20 μg PA21 protected all rats (Fig. 6b). Prophylactic function was tested by antibody injection at different times before LeTx injection. Even when PA21 was injected 24 h before LeTx, six rats survived (Fig. 6c).

### Protection mechanism of PA21

**Fig. 7:**
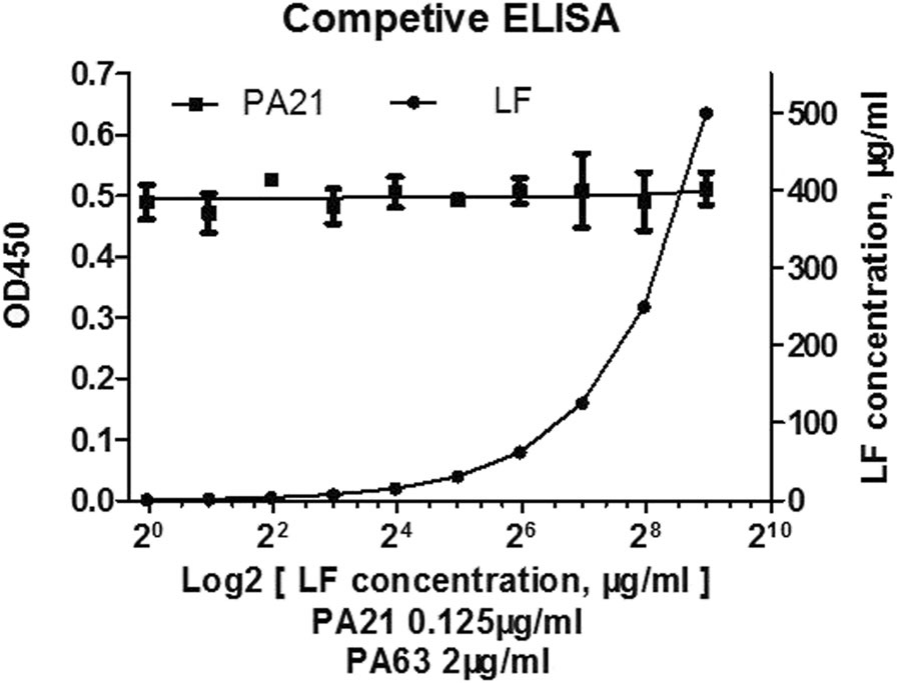
Competitive ELISA. PA63 was used to coat ELISA plates. The wells were then incubated with 0.125 μg/mL PA21 and serial dilutions of LF, and the bound antibody was detected by adding peroxidase-conjugated goat anti-human antibody followed by tetramethylbenzidine substrate. OD450 = optical density at 450 nm

**Fig. 8:**
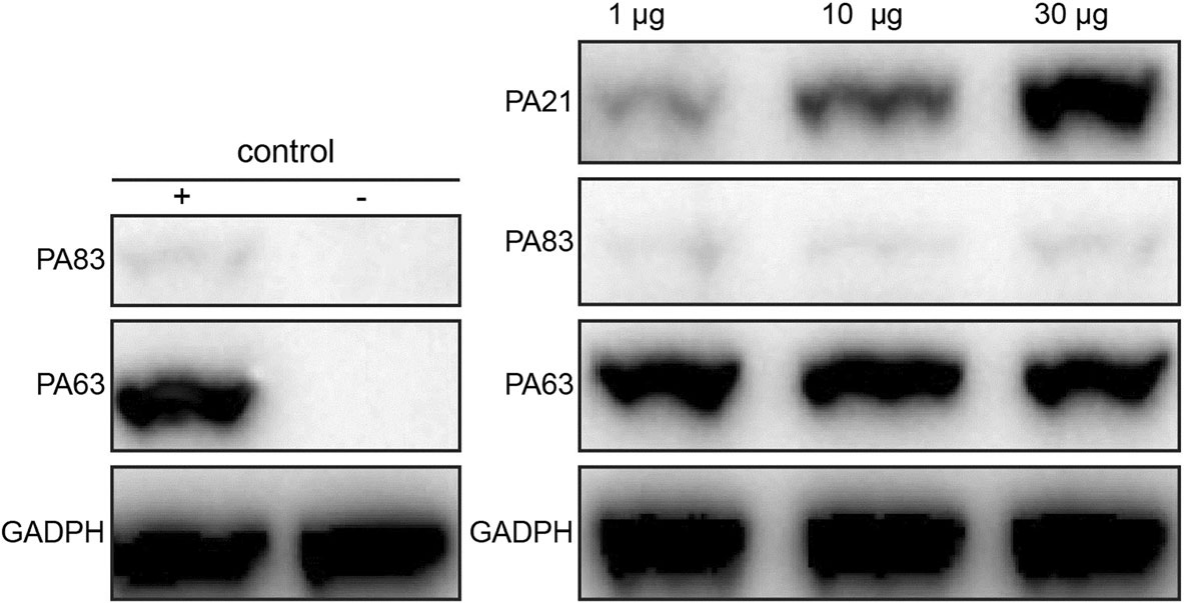
Western blot. J774A.1 cells were incubated with 1 μg PA83 and different concentrations of PA21 at 37 °C for 3 h in a 24-well plate. The cells incubated with only PA83 were used as the positive control, whereas the ones incubated with PBS were used as the negative control. Three concentrations of PA21 were used: 1, 10 and 30 μg

Competitive ELISA was performed with LF, PA21and PA63. PA63 was coated onto the wells of a 96-well ELISA plate at 2 μg/mL concentration. However, the OD_450_ was almost the same despite the increased LF concentration (Fig. 7). Then, the Western blot analysis in J774A.1 cells was performed. J774A.1 cells were treated with 1 μg PA83 and various concentrations of PA21. Therefore, with increased quantities of PA21, the detected amounts of PA83 and PA63 did not significantly change (Fig. 8).

## Discussion

From a human phage library, we recovered a Fab, which we then transformed to IgG that can bind PA with an affinity of 1.003 nM. The antibody PA21 neutralised the cytotoxicity of 10 μg/mL anthrax toxin at a concentration of 4 μg/mL in vitro. The molar ratio of PA21-to-PA is 0.2:1, which can efficiently protect animals from anthrax toxin challenging.

In previous studies, mouse mAbs and humanised murine mAbs have been reported [1, 19, 21]. These antibodies retained some antigenic components of the original non-human sequences and may elicit antibodies to the mAb in humans. Therefore, the ideal mAb for anthrax treatment or prophylaxis is a fully human antibody. However, few fully human anti-PA antibodies were tested in vivo. Amongst these tested antibodies, only two to three antibodies have been used in F344 rat models. Some of the anti-LF antibodies employed in F344 rats, for example, antibodies LF10E and LF11H [27], can confer 100% protection to F344 rats against LT challenge at the mAb-to-LF molar ratios of 0.5:1 and 1:1, respectively.

In our study, PA21 provided good protection in vitro test. PA21, with an affinity of 1.003 nM, protected all the cells at 1 μg/mL LF and 0.1 μg/mL PA83. By contrast, only 30% survival rate was observed for the non-conjugated IgG. PA21 protected more than 90% of the cells, whereas the control antibody protected only 26% of the cells in the presence of 10 μg/mL LF and 0.1 μg/mL PA83. Some human or human-like anti-PA mAbs have also been reported [1, 23, 24, 28, 29]. In the F344 rat model, PA21 provided better protective efficacy than these mAbs. For example, in Migone’s study, 1.5 mg/kg raxibacumab was administered 24 h before a lethal dose of anthrax toxin was administered to F344 rats [24]. However, the effective concentration of PA21 in the present study was approximately 0.14 mg/kg. In Chen’s study, the efficient W1-to-anthrax toxin molar ratio was 0.5:1 [28], whereas the PA21-to-anthrax-toxin molar ration the present study was 0.2:1. We also found a lower efficient mAb-to-anthrax-toxin molar ratio than that reported by Sawada et al. [23]. Furthermore, 0.14 mg/kg PA21 injected 5 min after the lethal toxin provided striking protection to F344 rats.

In a previous study, Abthrax, ETI-204, IQNPA and W1 [1, 24, 28, 30] all inhibited PA binding to cell receptors. In the present study, we found that PA21 and LF separately combined with PA63 and did not compete with PA63. The Western blot of the J774A.1cell lysate showed that PA21 did not inhibit PA83 binding to cell receptors or block cleavage of PA83 to PA63. Therefore, PA21 probably interferes with PA heptamer formation or disrupts the preformed PA heptamer by forming a super complex.

Anthrax is a constant threat to human health because of natural and bioterrorist-associated exposure. The passive administration of neutralising anti anthrax human mAb can provide immediate protection and emergency treatment for anthrax infection. PA21 holds the potential to be used in actual human treatment. Therefore, different animal models are needed to evaluate for further study. Moreover, future explorations of in-depth information on the mechanism of PA21 is necessary.

## Conclusions

In summary, we report a fully human IgG PA21 that can identify PA specifically with an affinity of 1.003 nM, exerts neutralization ability to LeTx in vitro and protective function in F344 rats in vivo. This fully human antibody PA21 can be used in anthrax treatment or prophylaxis in the future.

## Abbreviations

EF: Edema factor
ELISA: Enzyme-linked immunosorbent assay
ET: Edema toxin
F344: Fischer 344
IgG: Immunoglobulin G
LF: A lethal factor
LT: Lethal toxin
mAbs: Monoclonal antibodies
PA: Protective antigen
PAGE: Polyacrylamide gel electrophoresis
SDS: Sodium dodecyl sulphate
